# Citizens marine pilots: Increasing autonomous surface vehicles navigation capabilities with and for the public

**DOI:** 10.12688/openreseurope.21252.1

**Published:** 2026-01-12

**Authors:** Simona Aracri, Fausto Ferreira, Angelo Odetti, Giorgio Bruzzone, Marco Bibuli, Roberta Ferretti, Gabriele Bruzzone, Cristiano Cervellera, Massimo Caccia, Matko Batoš, Ivan Lončar, Juraj Obradović, Natko Kraševac, Vanja Asanović, Slavica Tomović, Žarko Zečević, Luka Martinović, Igor Radusinović

**Affiliations:** 1 Institute of Marine Engineering National Research Council, Rome, Lazio, Italy; 2 University of Zagreb Faculty of Electrical Engineering and Computing, Zagreb, City of Zagreb, Croatia; 3 University of Montenegro, Podgorica, Podgorica Municipality, Montenegro

**Keywords:** citizen science, marine robotics, AI, climate robotics, sustainable goals

## Abstract

Most of the world population lives by a water body. Observing, caring and understanding the ocean is vital for all. Among several branches of robotics - marine robotics creates the most interdisciplinary and participatory scientific pool, bringing citizen and scientists close to the use of aquatic robots. International efforts, such as the EU-funded MONUSEN project, are pioneering the technological transfer across borders, disciplines and people, therefore sustaining the United Nations Sustainable Development Goals (Goal 14 Life below Water). In particular, under MONUSEN we carried out two activities involving the general public, demonstrating the use and utility of cost effective technology. The first activity saw the presentation and usage of MARUS - Marine Robotic Unity Simulator - which offers advanced capabilities of generating realistic maritime environments allowing for closer-to-reality V&V of applications developed for maritime vehicles. The second activity shared the functionality of the autonomous surface vehicle (ASV) SWAMP (Shallow Water Autonomous Multipurpose Platform). Participants piloted SWAMP ASV, collecting data along a predetermined path. Through the active involvement in data collection, they gained knowledge of state-of-the-art methods for controlling autonomous surface vehicles and contributed to a learning-by-imitation experiment. Both activities showed that the setting of the experiments and the visual interfaces effectively engaged a wide range of people belonging to different ages, cultures and education. The activities transferred new knowledge to the public, truly bringing them closer to the scientific work carried out in MONUSEN.

## Introduction

It is now more important than ever to disseminate scientific findings to the general public and actively involve citizens. In an era of climatic instability, offering solid insights in the results of research fosters trust in the scientific process. Furthermore, the participation of the wider public in the data collection and interpretation brings the academic world closer to the citizens’ needs, thus driving the technological advances towards the necessities of society.

The MONUSEN project ^1^ aims at enhancing the capacity and resources of University of Montenegro Faculty of Electrical Engineering. It targets excellence in Underwater Sensor Network (USN) communication protocols and security, USN data processing, and mobile USNs. The project focuses on developing energy-efficient, secure, and reliable USNs for long-range marine exploration.

MONUSEN embraces citizen science initiatives in addition to press releases, newsletters, website updates, and social media presence. General public engagement ^2^ plays a vital role in improving transparency in scientific research and fostering trust in scientific projects. When citizens actively participate in the scientific process, they share their knowledge, observations, and experiences. By doing so, we not only promote the understanding of research in the field of USNs, but also showcase its real-world environmental applications. In this case, outreach events and activities significantly raise awareness about the MONUSEN project and its impact on society, bringing the world of USNs closer to citizens. This approach also stimulates interest and trust in science not only in the country hosting the activity but also across our partner countries.

Citizen science aims to raise awareness about the Sustainable Development Goals of the United Nations (Goal 14 Life below Water) by explaining what can be achieved with USNs in the field of environmental protection and climate change. The engagement of the general public has in recent years seen considerable growth thanks to web-based and mobile platforms and technological advancements. In addition to serving as a tool for raising awareness, education, scientific literacy, and improving scientific communication ^3^, it allows for expansive data collection creating large longitudinal data sets suitable for biodiversity monitoring and marine-based research ^4, 5^, forming, for example, an early response to newly established populations thanks to surveillance and monitoring of invasive species ^6^, and, in general, forming a quantitative understanding of habitat and climate shifts as they happen ^7^. Work is ongoing on the integration of citizen science approaches into the Sustainable Development Goals (SDGs) of the United Nations (UN), with analyses that attempt to pinpoint the greatest benefits of the input that citizen science can provide to the SDG framework ^8^ and roadmaps describing ways to integrate citizen science into the formal reporting mechanisms of the SDGs ^9^. These factors strongly motivate our decision to integrate citizen science activities into our project. Using citizen science, our aim is to engage the public in meaningful ways, fostering a sense of ownership and collaboration in achieving these global goals. Through these activities, we plan not only to raise awareness of the UN SDGs, but also to empower people to actively contribute to the preservation of our oceans and the sustainable development of our economies. To achieve the goals above, experiments and activities dedicated to the general public happen, typically during summer schools and large events, within MONUSEN project.

In recent times, the use of autonomous robotic vehicles in less structured environments and their integration into everyday human activities has given rise to a range of legal and societal challenges concerning public acceptance. This, in turn, has significantly hindered the uptake of robots in comprehending unstructured settings marked by intricate dynamics and interactions ^2^. Furthermore, the operation of autonomous marine vehicles currently lacks regulation by the International Maritime Organization. This absence of regulatory oversight, coupled with societal barriers and misconceptions, is hampering the integration of unmanned/autonomous robots in civil and commercial domains. This predicament is largely due to the public’s perception of ports, shipyards, and marine technology as sources of heavy pollution rather than engines of eco-sustainable technological advancement. To alter this perception, it is essential to disseminate research results in this field to the general public. On the other hand, the European Community is actively promoting citizen engagement as an effective mean of bridging the gap between the public, experts in the field, and policy makers ^10^. Consequently, involving citizens can help foster social acceptance of autonomous robots as integral components of our daily lives.

Section 1 describes the citizen science experiment held in Limerick during OCEANS 2023. Section 2 describes the activities that we carried out in Kumbor during Breaking the Surface BtS-2023. Each experiment is presented with its own narrative. Section 1 is divided into Rationale, Methods for Engagement and Results. Section 2 is divided with Rationale, Methods for testing, Experiment description, Results. Ultimately we report our final remarks in the Conclusion.

## Citizen science at OCEANS 2023 - Limerick

The first citizen science activities took place during OCEANS 2023 conference from 5th to 8th June 2023 in Limerick, Ireland ^1^. During OCEANS 2023, two complementary activities took place. A hands-on tutorial using the Marine Robotic Unity Simulator (MARUS) simulator ^11, 12^ was performed on the first day of the conference. In addition, a MONUSEN booth in the exhibition space engaged a wide range of different people in the project’s activities. Citizens could test the MARUS simulator and participate in a citizen science experiment both during the tutorial and by visiting the booth. Below we describe the MARUS simulator and the tutorial delivered to the participants.

### Rationale

Research in marine robotics requires frequent use of simulators due to the complexity of trials which usually happen in the water. During OCEANS 2023 Limerick, We presented our high-fidelity simulator MARUS that offers advanced capabilities of generating realistic maritime environments allowing for closer-to-reality V&V (Verification and Validation) of applications developed for maritime vehicles. The simulator offers synthetic dataset generation with perfect annotations for various sensors (cameras, LiDAR, sonar) and allows for interaction with the environment for closed loop simulation. This simulator is highly applicable for the researchers in the field of marine robotics in industry and academia and it has been used in a myriad of applications such as diver-robot interaction, unmanned ships etc ^13, 14^. MARUS simulator is built in Unity Engine as it provides tools for generating realistic environments and easy to use interface for quick setup which makes it a perfect tool for development of a simulator. The public received a deep dive tutorial into MARUS by its creators - the Laboratory for Underwater Systems and Technologies (LABUST) from the University of Zagreb Faculty of Electrical Engineering and Computing, one of the MONUSEN partners. The tutorial introduced all the tools used for the development and characterization of the simulator. Features include many different types of sensors, thruster simulation, tools for data annotation, connection with the Robotic Operating System (ROS) 1 or 2 middleware ^15, 16^ simple controls etc. During the practical part of the tutorial the participants assembled a vehicle in a pre-built marine environment and equipped it with different sensors such as cameras, LiDAR, Inertial Measurement Unit (IMU), GPS, etc. In addition, the audience was introduced with ROS and in particular with the sensor data available in ROS topics. The second practical part involved the use of annotation tools that can be useful for solving situational awareness tasks. We showed annotation tools for different sensors developed in the simulator. Participants also performed a Citizen Science activity where they completed a task in the simulator environment.

### Methods for engagement

In addition to the tutorial, the MONUSEN booth included a PC with the simulator installed and ready to be easily used by any by-passer. Visitors were briefly introduced to the task needed to be completed and asked to participate in the experiment. In addition, after completing the activity they were invited to complete the survey online. Unfortunately, collecting good data in these conditions was not always easy as attendees have a limited time to visit the exhibition area and might not stay until the end of the experiment and/or not all participants in the experiment actually filled out the survey. These are limitations that affected the number of completed surveys and experiments. Nevertheless, performing this experiment at OCEANS 2023 Limerick allowed us to explain the need of efficient autonomous navigation to the attendees and to show our work to a very broad international audience, which gave us a good qualitative feedback. Informed consent was sought and obtained for each individual that participated in the OCEANS 2023 Limerick activities.

In the citizen science experiment, participants engaged in a Unity game where they controlled the MARUS boat, navigating through various maritime scenarios to avoid collisions with other ships. The game featured five levels, each progressively more challenging than the last. Initially, participants were briefed on the International Regulations for Preventing Collisions at Sea (COLREGs) to ensure they understood the fundamental rules of maritime navigation ^17^. Potential collision situations are separated into five scenarios: Overtaking, Head on, Right crossing, Left crossing and Head on - Right crossing. In Level 1, players faced an overtaking scenario ^18^, where they needed to overtake a slower boat directly in front of them to reach their goal. Level 2 introduced the head-on scenario ^19^, requiring players to avoid a boat moving towards them. Level 3 focused on right crossing ^20, 21^, a straightforward scenario similar to earlier levels, but requiring precise manoeuvring. Level 4 was more complex, involving a left crossing ^20^ where the target ship did not adhere to COLREGs ^22^, challenging players to navigate safely despite the non-yielding ship. The final level combined the challenges of head-on and right crossing scenarios, testing players’ ability to handle multiple potential collision situations simultaneously. This experiment aimed to gather data on how well participants could apply COLREGs in simulated environments and improve their understanding of maritime navigation through an interactive gameplay. The same collision situations are tested with an autonomous ship and autonomous collision avoidance algorithms. The additional goal is to compare the efficiency of citizens’ choices and algorithm choices and inform citizens of the need to be both safe and efficient to avoid collisions at sea and to save fuel, thus saving energy and the environment.

### Results

**Figure 1.**
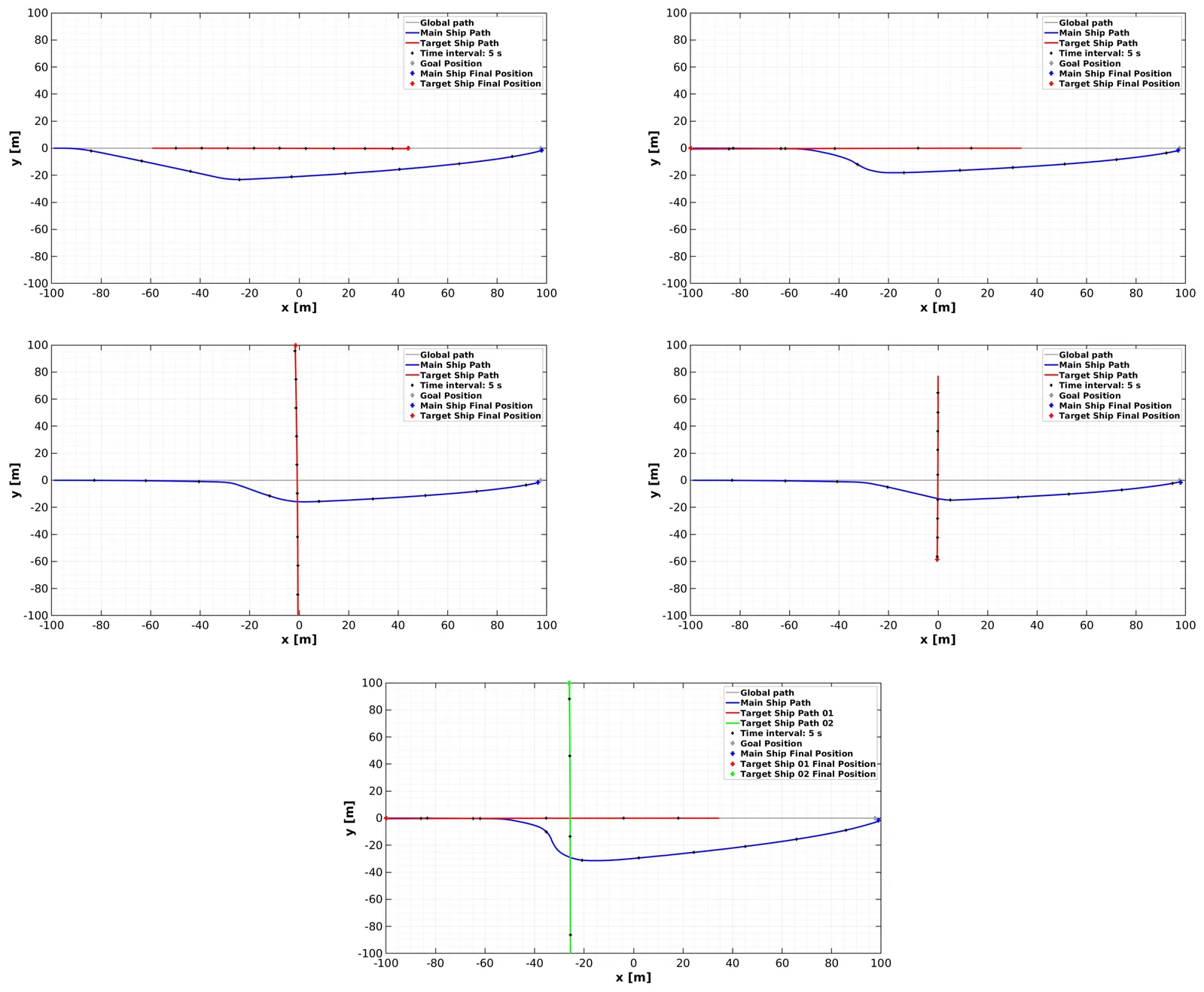
Path of the target ship and the main ship in the following scenarios (left to right): Overtaking, Head on, Right crossing, Left crossing, Head on - Right crossing. The main ship is controlled with the autonomous collision avoidance algorithm.

Not all citizens successfully completed all 5 levels due to the abovementioned reasons. Nonetheless, here we present quantitative results based on 12 attendees. Figure 1 shows the paths of the main ship, for the 5 different scenarios, when controlled by an autonomous collision avoidance algorithm.

**Figure 2.**
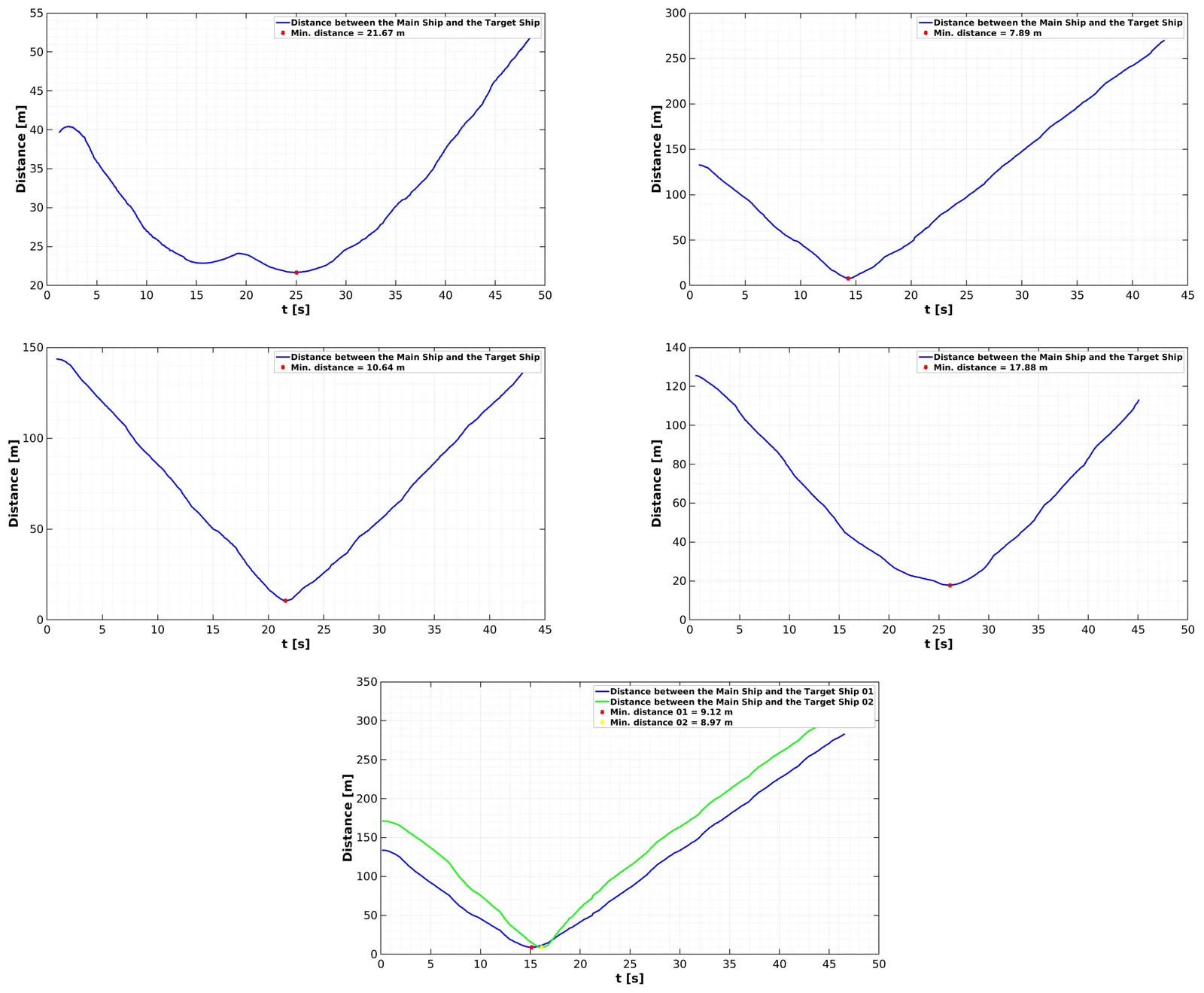
Distance between the main ship and the target ship. Plots are in the same order as in 1.

**Figure 3.**
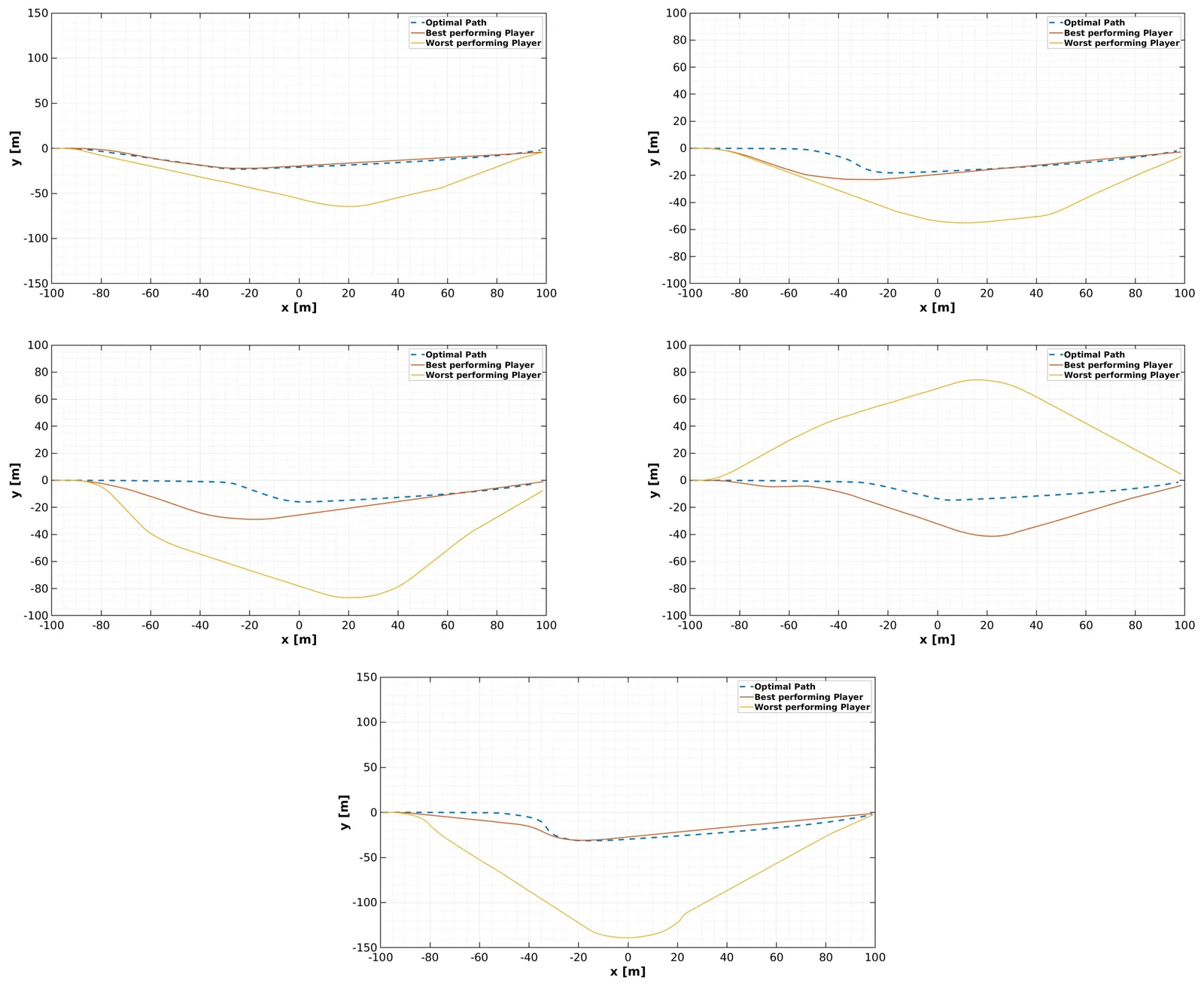
Path of the main ship of the players with best and the worst results in comparison with the main ship controlled with the collision avoidance algorithm. Path provided by the collision avoidance algorithm is considered optimal. Plots are in the same order as in 1.

**Figure 4.**
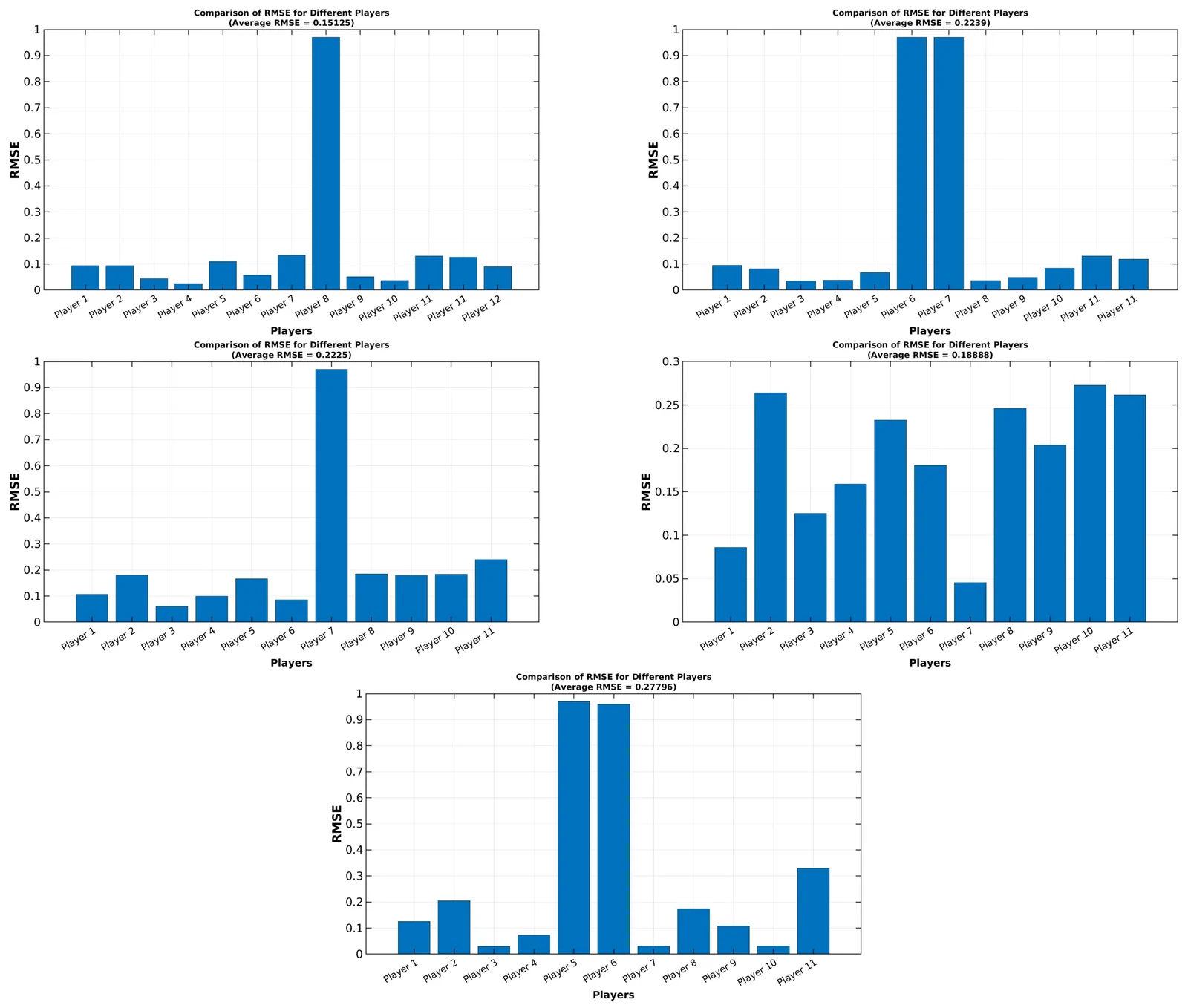
RMSE of all the players participating in the citizen science activity. Players with the RMSE value close to 1 collided with the target ship. Plots are in the same order as in 1.

**Figure 5.**
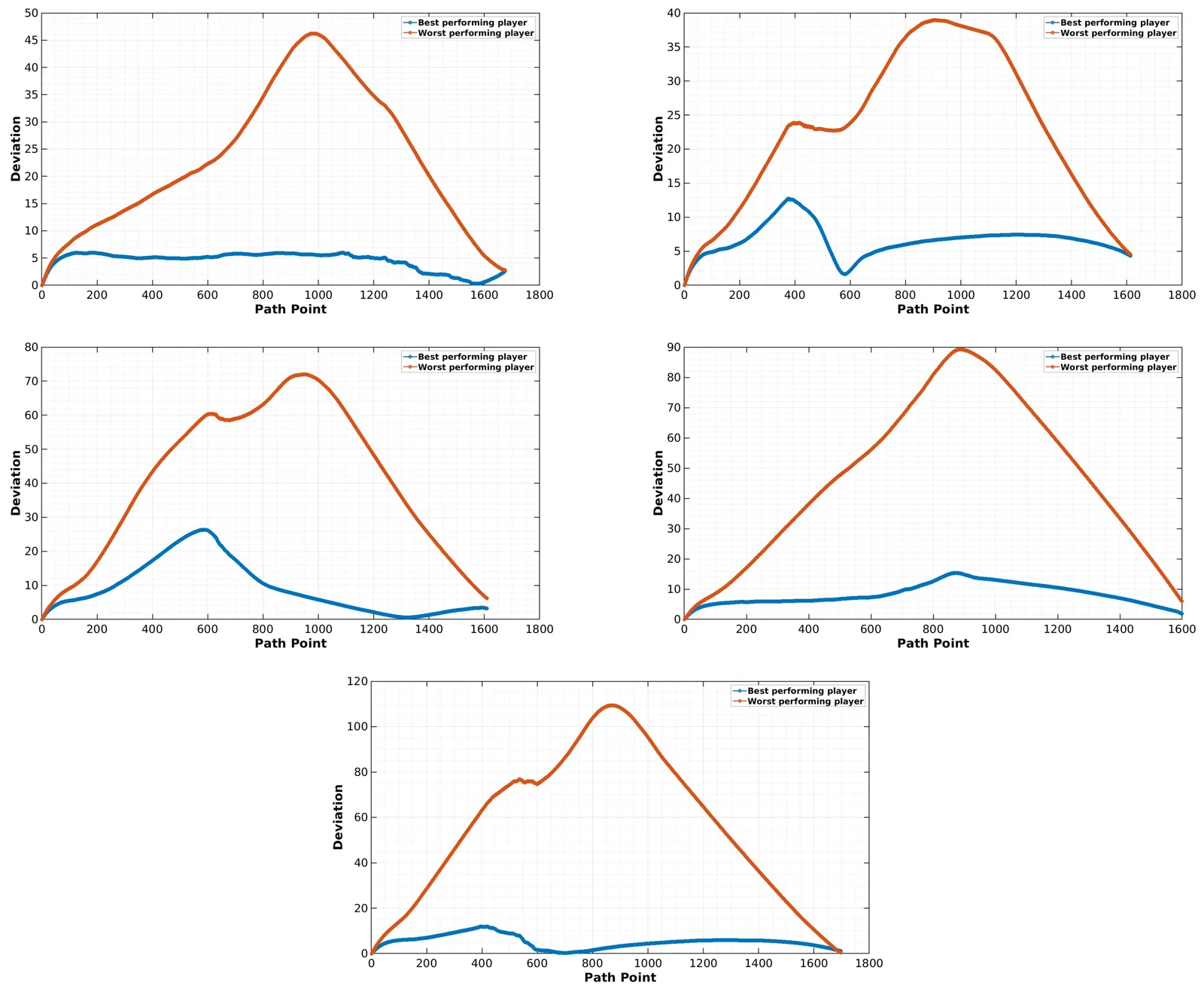
Deviation of the best and the worst performing players from the optimal path. Plots are in the same order as in 1.

**Figure 6.**
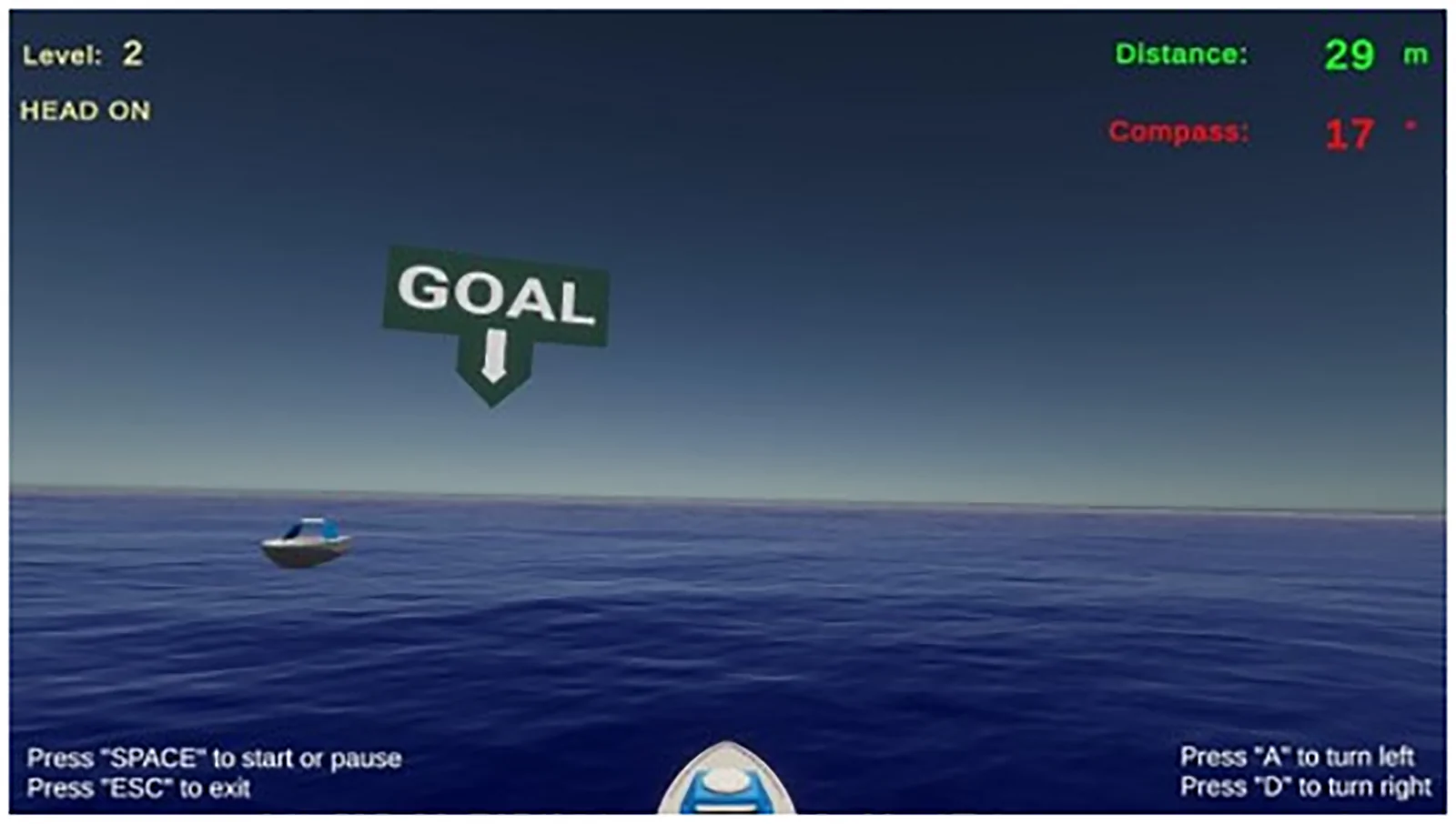
An example of the Head on scenario from the citizen science activity.

Figure 2 presents the corresponding distances between the main ship and the target ships for the case when the main ship is controlled by the autonomous collision avoidance algorithm. Figure 3, Figure 4 and Figure 5 indicates the best and the worst performing player results, as well as normalized RMSE (Root Mean Square Error) values and deviations from the optimal path calculated by the algorithm. An example of the Head on scenario from the citizen science activity is shown in Figure 6. The use of citizen science in this context provided valuable insights into public awareness and adherence to maritime safety rules. Looking at the average of normalized RMSE, the values range between 0.15125 and 0.27796, with the lowest value in Level 1 and the highest value in Level 5, which was to be expected due to the increased level of difficulty.

Through a survey we gathered the participants’ feedback on the experiment. We also fostered discussions during the tutorial and welcomed qualitative feedback during the interactions with visitors of the booth. As in previous similar user experiences ^23^, the survey included a first part with background questions regarding the previous experience of attendees with boat driving followed by a second part dedicated to task load evaluation. It is important to understand if the simulator can be used as a training tool for potential boat captains. Thus, the NASA Task Load Index (TLX) developed by Hart and Staveland ^24^ was used to evaluate the subjective mental workload with indexes of mental, physical and temporal demand, performance, and difficulty levels. The five components were rated from 1 to 10. Finally, the third and fourth parts of the survey addressed qualitative feedback regarding the simulator and the experiment itself and asked for suggestions on future developments of the simulator.

Twenty participants provided complete answers to the questionnaires of which 16 attendees of the tutorial and 4 booth visitors. 45% of the participants were between 18 and 30 years old. 25% of the respondents were between 31 and 40 years old. Out of the 20, 10 did not have any previous boat driving experience, 5 occasionally engaged in boat driving, 2 had a medium level of experience, 2 an advanced level (e.g. part of their jobs) and one did not answer. Only a quarter of the attendees (5 people) had experience of developing simulations or games and the average first-person-view gaming skills was 5.65 out of 10.

When it comes to the NASA Task Load Index (TLX) estimation, as mentioned, 5 indexes were used: mental effort (ME), physical effort (PE), time pressure (TP), performance (PF) and task difficulty (TD). On a scale of 1 to 10 (0 no effort, 10 impossible), ME average was 4.26 while PE was only 2.74. This is expected as pressing buttons is not physically demanding, but the mental effort required (especially for non-specialists) is higher than the physical effort. In terms of time performance (amount of pressure felt due to the rate at which the task elements occurred), the average value was 4.11 (still below half of the scale). On the other hand, participants rated their performance successful on an average of 5.11 (above half) with a standard deviation of 2.81. This large standard deviation and half-success rate can be explained by the fact that the sample was not broad enough (20 participants from different backgrounds) and all missions were considered together when answering this question (both the more advanced levels and the easier ones). This is corroborated by the fact that the average value for task difficulty was 4.53 (10 = difficult, 1 = easy).

Looking into the feedback about the simulator itself, the median value of realism was 7 (from 1 to 10 as well). 70% of the players classified the 5th level as the hardest as expected as this was the level involving more boats, while 88% declared that their longest trajectory was on the 5th level. Attesting to the lack of experience, 50% of the attendees was not aware of COLREG rules at all before the game. 20% instead knew all the COLREG rules involved and 30% knew just a few. The median value for the potential of using this simulator for boat driving training was a high 8 (from 1 to 10) with 55% of the players choosing 8 or above, which shows the goodness of the MARUS simulator for this training task. Similarly, 65% of the attendees chose 8 or above (out of 10) to rate the potential of using situational awareness modules to help humans follow COLREG rules in Marine Traffic. When asked about the biggest drawback of the simulator, several attendees identified the lack of a wide field of view/lateral vision, while final comments were positive and encouraging, highly appreciating the simulator.

## Citizen science activities in breaking the surface 2023 - Kumbor

The citizen science activities of the second year took place during the Summer School in Kumbor, Montenegro, i.e. the 15th Breaking the Surface (BtS) International interdisciplinary field workshop on maritime robotics and applications ^25^ from September 24th to September 30th, 2023. The MONUSEN project coordinator, Faculty of Electrical Engineering at the University of Montenegro, in collaboration with the partnering institution, the National Research Council of Italy – CNR, organized a demonstration workshop on September 27th 2023, in Kumbor, Montenegro. This demo workshop was held under the MONUSEN project and “The Involvement of Citizens in the Scientific Activities of the MONUSEN project - CSI MONUSEN” under the patronage of Montenegro’s Ministry of Science and Technological Development.

### Rationale

Students and teachers from High School ‘Mladost’ in Tivat and Gymnasium Kotor - Montenegro - participated in the demo workshop as ‘citizen scientists.’ Project members presented their research in the area of underwater sensor networks during the workshop. The session included an explanation of the construction and functionality of the autonomous surface vehicle SWAMP (Shallow Water Autonomous Multipurpose Platform) ^26^. Students collected data by piloting SWAMP remotely. Such data trained the controller for automatic vehicle control. The students also received insights into various methods for automatic vehicle control, methodologies for collecting and visualizing data, and performance evaluation of the vehicle in task completion. To promote research activities in the field of underwater sensor networks, promotional materials such as t-shirts, notebooks, and flyers were distributed among the students, along with Certificates of Participation. The CSI MONUSEN workshop aimed to highlight the importance of new technology in addressing environmental challenges along the coast and sea. Through active involvement in data collection, participants gained knowledge of state-of-the-art methods for controlling autonomous surface vehicles. Twenty-five high school students participated in data collection as “citizen scientists” by manually controlling the autonomous surface vehicle. The purpose of involving high school students is to promote science and technology, encourage creative thinking, support young talents, and contribute to developing a knowledge-based society.

### Methods for testing

For activities involving underage students, parental or guardian consent was obtained prior to any photo or video recording conducted during the CSI–MONUSEN events. The consent forms explicitly authorized the use of such materials for pedagogical purposes (e.g., documentation of student activities and achievements, presentation of work by students, associates, and faculty, and professional development) and for promotional purposes related to the Faculty of Electrical Engineering of the University of Montenegro and the MONUSEN project (e.g., billboards, electronic and printed materials, academic publications, the faculty website, social media profiles, and press releases). As the activities were limited to outreach and educational actions within the CSI–MONUSEN project, no separate ethical approval number was issued by an institutional ethics committee.

**Figure 7.**
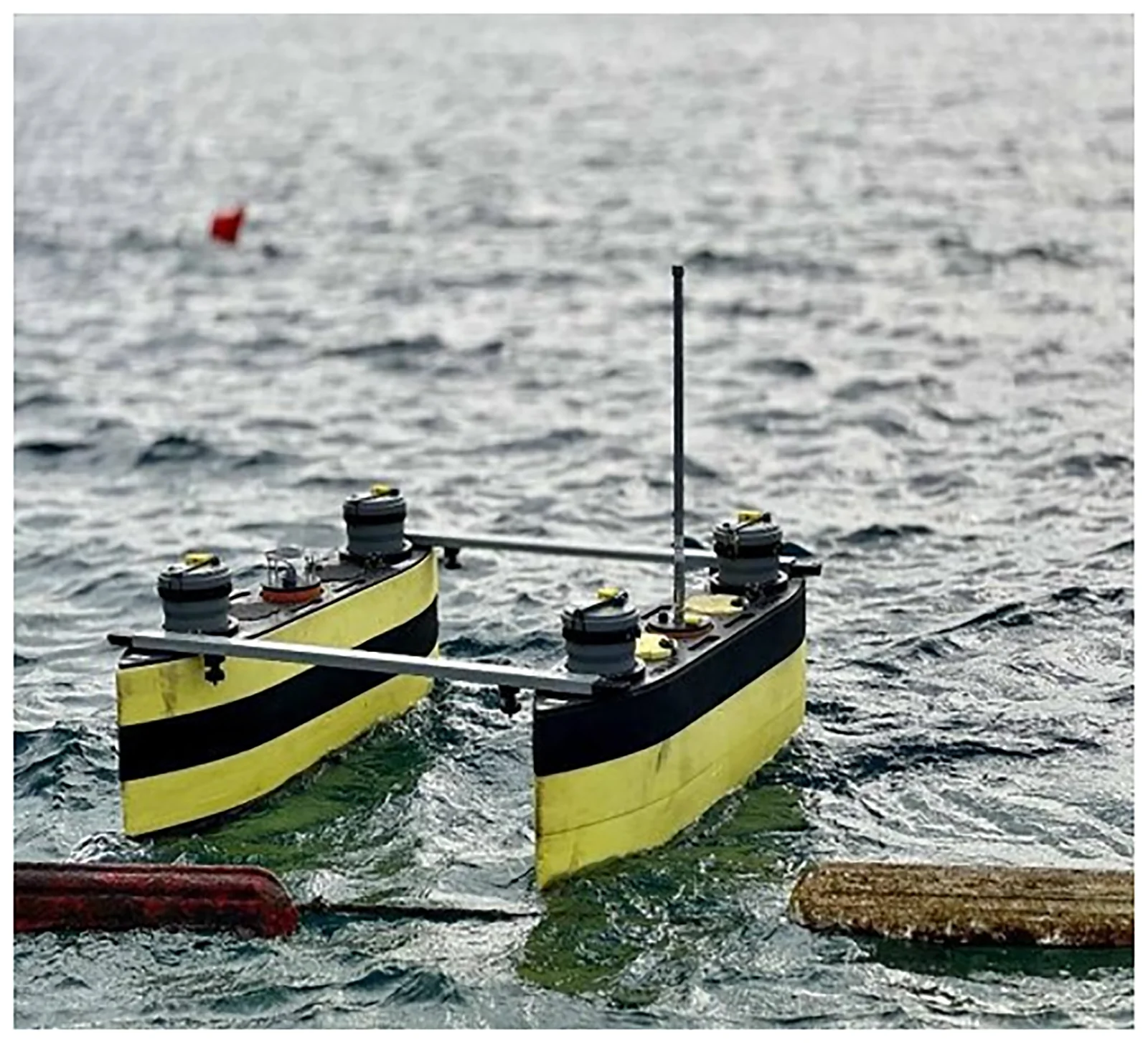
SWAMP (Shallow Water Autonomous Multipurpose Platform).

The main objective was to demonstrate to participants the principle of controller design for Unmanned Surface Vehicles (USV) based on the imitation learning paradigm ^27^. In contrast to conventional controller design methods that rely on knowledge of the vehicle model, the imitation learning paradigm bases controller design on collected data. Typically, the robot/vehicle is manually controlled to complete a specific task, and during the experiment, all data is collected. Furthermore, based on the collected data, the neural-network (NN) controller with a predefined architecture is trained to imitate human actions. Specifically, in Kumbor, the imitation learning paradigm is employed to control the SWAMP platform shown in Figure 7. Four buoys were positioned at predetermined locations in the sea, in front of the coast of Hotel Carine in Kumbor. The SWAMP was developed and assembled at the Institute of Marine Engineering (INM), National Research Council (CNR) laboratories located in Genoa, Italy. The sea trial was structured to actively involve citizens in data collection by allowing them to steer the vehicle remotely, thus contributing to the research efforts. The vehicle weighs approximately 40 kg and has dimensions of 1.25 m in both length and width, with a height of 0.5 m. Its draft is 0.12 m. The design of SWAMP incorporates a soft structure made from Polyethylene soft foam, which, combined with its propulsion system based on flush-integrated Pump-Jet Thrusters ^26^, ensures safe operation even in densely populated environments, such as areas filled with swimming tourists. The use of PE foam minimizes the risk of injury upon contact, while the flush design of the propulsion unit eliminates the exposure of moving parts, further enhancing safety. These features not only protect individuals coming across SWAMP, but also foster public trust and acceptance of robotic technology in recreational or leisure environments, where safety is often the highest priority. As a result, the SWAMP robot is well-suited for deployment in sensitive areas, such as beaches and swimming pools, helping to bridge the gap between advanced technology and public safety concerns.

Before the CSI-MONUSEN workshop, preliminary testing and project preparations occurred on September 25th and 26th, 2023.

### Imitation learning control paradigm

In order to implement an imitation learning control scheme, each task demonstration from the human controller has been recorded at discrete time steps. Specifically, the variables recorded at each time step k are *x(k)*, *y(k)*, *ϕ*( *k*), joythrust(k) and joyangle(k), i.e., the position and angle of the robot and the corresponding given joystick inputs ^26^.

**Figure 8.**
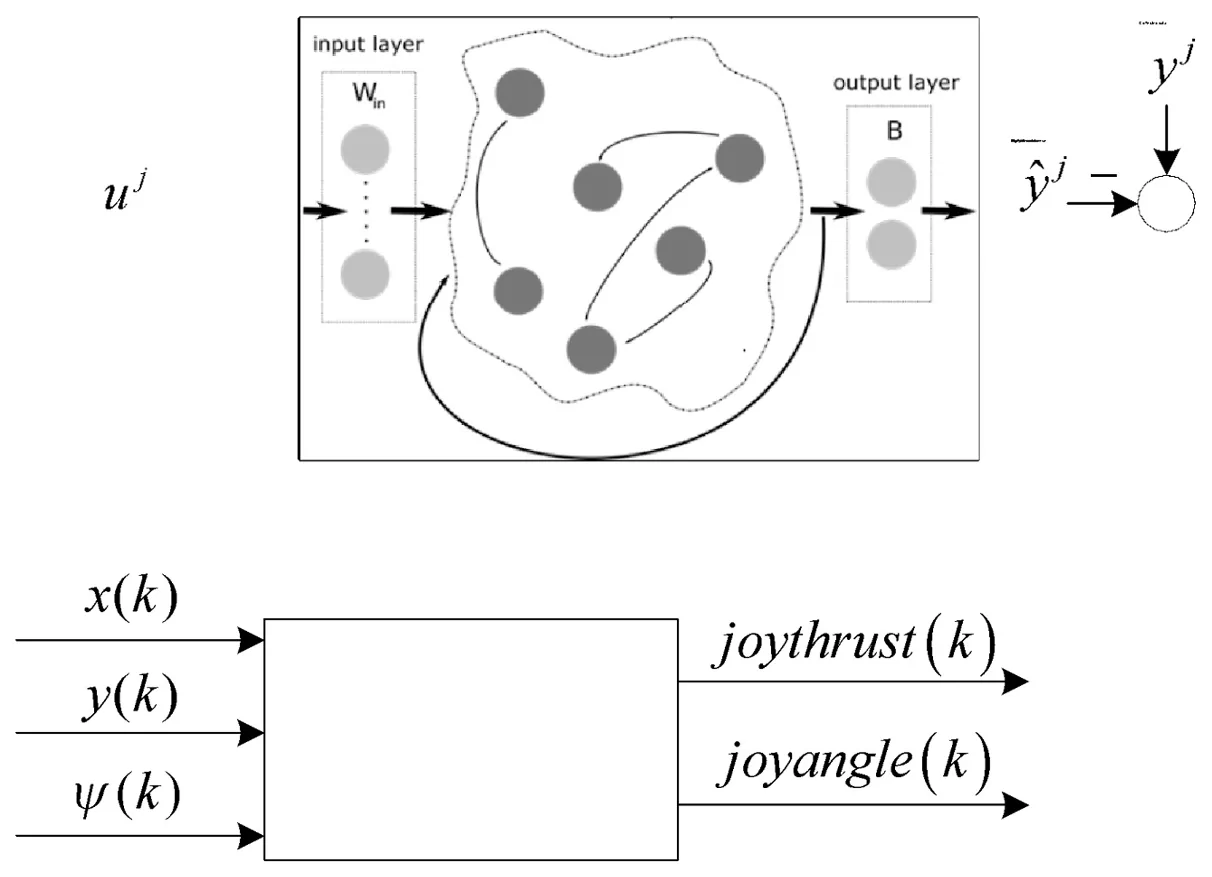
Imitation learning control paradigm.

From each trial, the datasets *u ^i^* = { *x*( *k*), *y*( *k*), *ϕ*( *k*)} and *y ^i^* = { *joythrust*( *k*), *joyangle*( *k*)} for *k* = *0*, *K*, *K ^j^* are collected, where *j* denotes ordinal number human operator and is the total number of steps composing the trajectory. The collected data is used to train a neural network controller, with *u ^j^* serving as the input to the controller and *y ^j^* as the desired output. In particular, due to the dynamic nature of the application, a recurrent neural network having the form of an Echo State Network (ESN) has been employed. For a detailed description of the neural controller and the training procedure, the reader is referred to ^26^. During the training process, the coefficients of the neural network are optimized to approximate the relationship between joystick inputs and the position and angle of the robot, as illustrated in Figure 8. Following the training phase, the acquired neural network is employed to automatically control the SWAMP based on real measurements. In other words, the NN controller uses actual measurements of the robot’s position and angle to generate outputs that correspond to joystick inputs, enabling the USV to perform tasks for which the neural network has been trained ( Figure 8).

### Experiment description

**Figure 9.**
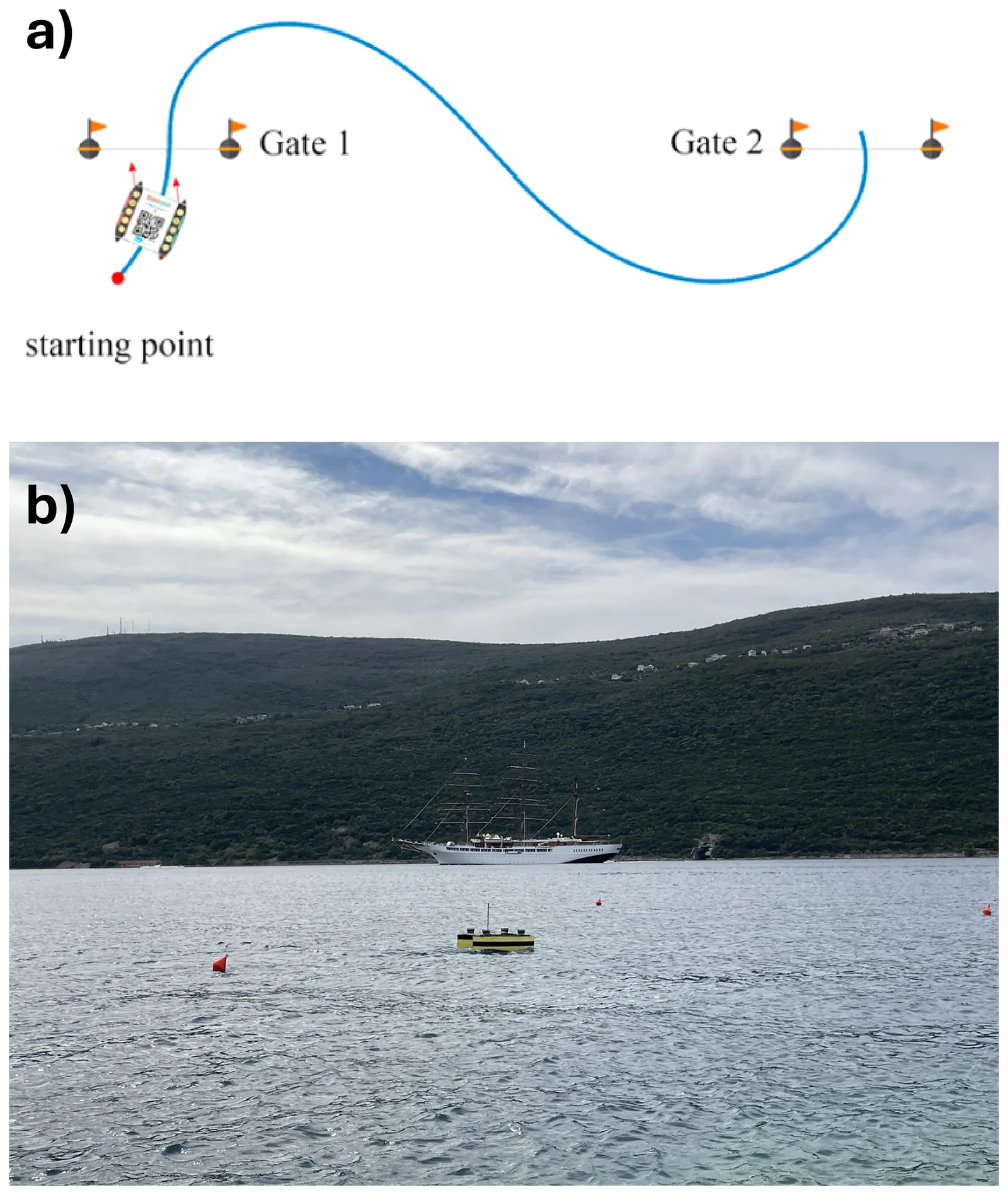
Experimental set up for the Citizen Science activity in Kumbor. **a**) illustration of the S-shaped path; **b**) arrangement of the buoys indicating the two gates through which the students piloted SWAMP.

Following a brief demonstration and instructional session, students were tasked with manually operating SWAMP. Specifically, students used a Thrustmaster USB Joystick to pilot the SWAMP through a course signposted by two gates, formed by four buoys, creating an S-shaped path ( Figure 9).

The vehicle’s trajectories and control inputs were recorded and used to train a neural network using the imitation learning paradigm. The objective of this training was to train an AI controller to autonomously drive SWAMP. Students proactively engaged with remotely controlling the robot, for some the experiment has been perceived as playing a video game.

## Results

**Figure 10.**
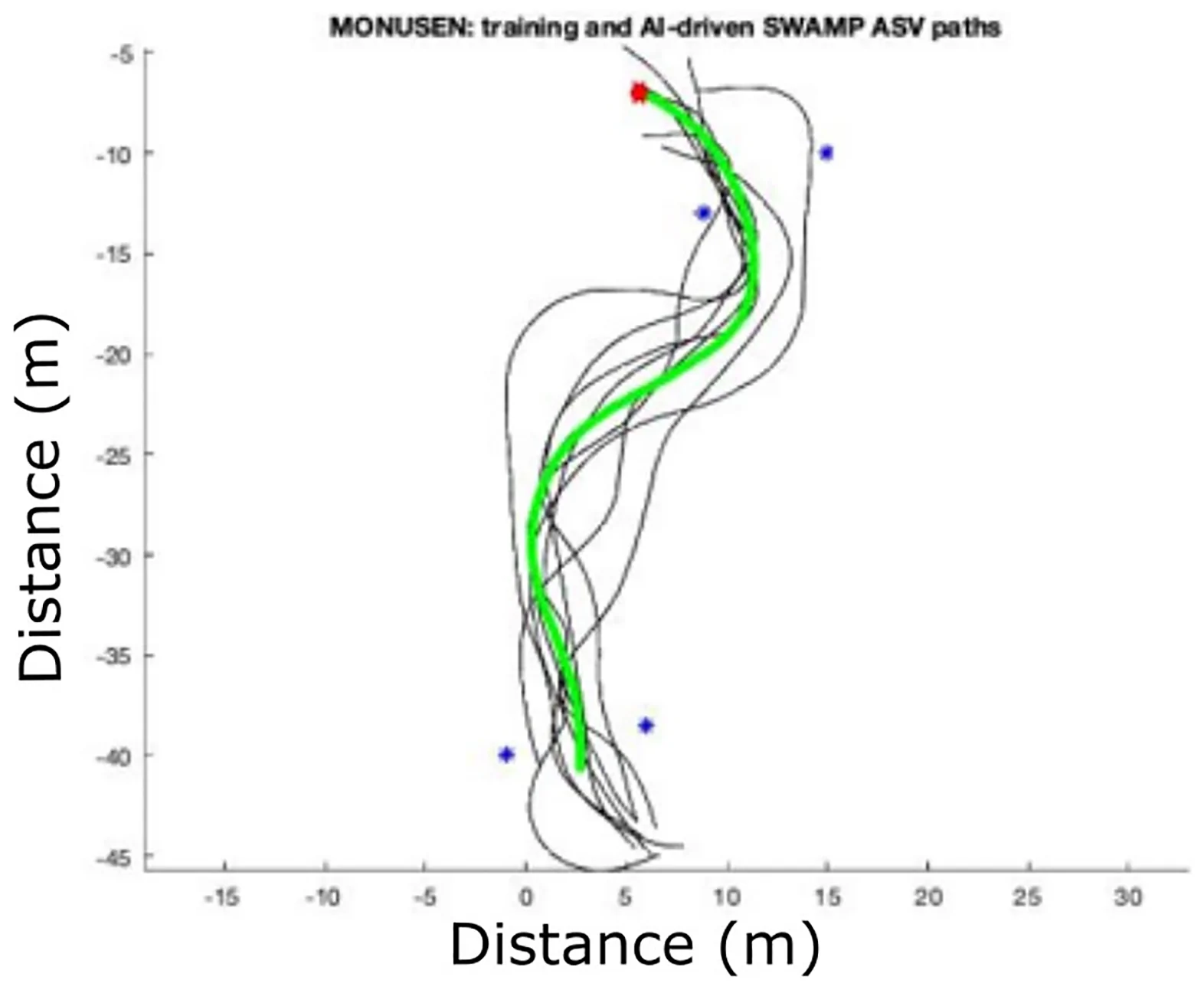
Subset of training path (black) and AI-driven path (green).

CNR team in Genoa fine-tuned the parameters of the neural network controller. Subsequently, the AI controller was tested in fully autonomous control of SWAMP, without any human intervention. A subset of training trajectories is plotted in black in Figure 10, where a trained AI-driven path is represented in green (the red asterisk marks the trajectory starting point). Blue asterisks mark the position of the buoys.

**Figure 11.**
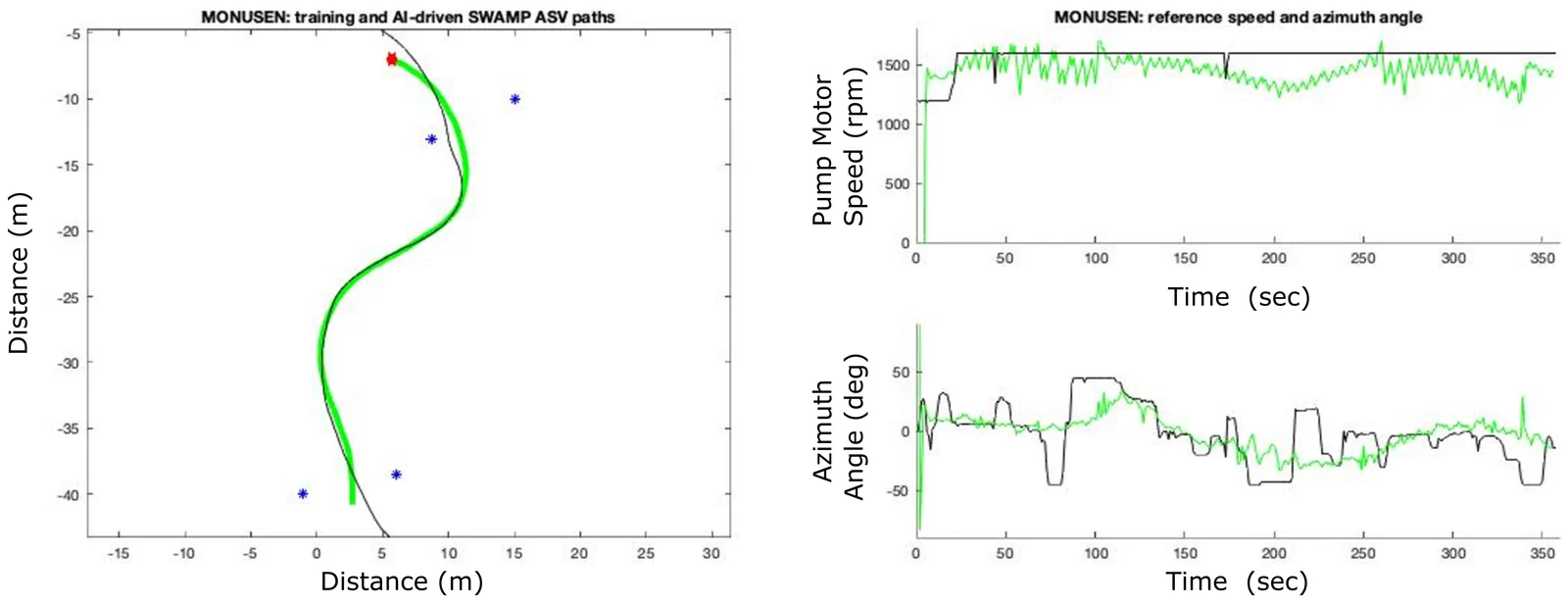
Left: an example of training (black) and AI-driven paths. Right: reference pump motor speed [rpm] (top) and azimuth angle (bottom). Black: human pilot; green AI pilot.

Figure 11 depicts the reference pump motor speed in rpm and azimuth angle in degrees provided by the human pilot and the trained AI controller. A trajectory executed by a skilled pilot has been considered. The executed paths are very similar. The human pilot maneuvered the vessel at constant maximum speed, while the AI-pilot slightly changed it (top plot). The smoothness of the AI-pilot is more evident when considering the reference azimuth angle, commanding the ASV turning. As shown in the bottom picture, the human pilot used a kind of bang-bang strategy with respect to the smooth control action executed by the AI-driver.

## Conclusions

The use of digital twins in the experiments allowed representation and interactions of the public with real robots. The cognitive clarity of the digital twins invites for a fast and proactive simulated experience.

This is the case of the MARUS simulator which can provide an experience with several surface and underwater vehicles as well as divers. The user’s experience in Limerick produced perceptive results through NASA Task Load Index and a questionnaire. We extracted mental effort, physical effort, time pressure, performance and task difficulty. Revealing a positive experience overall, a relatively low stress for the individuals. This shows the possibility of using the MARUS simulator for digital twins of boats/ships to train captains and amateurs on piloting those boats/hips.

The data collected by the students when piloting SWAMP can be embedded in a digital twin of the ASV. The experiment of citizens engagement in robotics operations, focusing on the imitation learning paradigm and AI technology. In a healthy, competitive, atmosphere, students gained an understanding of the importance of involving citizens in scientific research, artificial intelligence, and marine robotics as a new paradigm in research and innovation. They learned about the significance of developing autonomous surface vehicles for acquiring and monitoring environmental parameters in conditions that can be unhealthy and dangerous for humans. The positive response from students, coupled with their active participation in steering the SWAMP during the sea trial, not only contributed to valuable data, but also played a crucial role in fostering social acceptance of autonomous robots. In particular, the direct participation of citizens in robot training by imitation may serve as a practical mean to build public trust in robots performing autonomous operations in crowded areas, and a clear knowledge and understanding of the rules under which they operate. From a technical viewpoint, the collected data will be used for further research in this field, which involves exploring different network architectures and more complex imitation learning paradigms, investigating the issue of selecting appropriate training trajectories, and exploiting the integration of simulated and real-world experiments.

Overall, this research underscores the potential of integrating robot soft design, digital twins, AI, and citizen engagement in advancing autonomous robotics, paving the way for safer, more efficient, and socially accepted robotic systems in the future.

## Ethical approval statement

On behalf of the University of Montenegro - For activities involving underage students, signed parental/guardian consent was obtained for photo and video recordings during the CSI–MONUSEN events. These consents explicitly covered the use of recordings and images for pedagogical purposes (e.g., documenting student activities and achievements, presenting the work of students, associates, and professors, and professional development) as well as for promotional purposes of the Faculty of Electrical Engineering of the University of Montenegro and the MONUSEN project (e.g., billboards, electronic and printed publications, academic papers, the faculty website, social media profiles, and press releases). As the study consisted solely of outreach and educational activities conducted under the CSI–MONUSEN project framework, no separate ethical approval number was issued by an institutional ethics committee.

On behalf of the University of Zagreb, the Faculty of Electrical Engineering and Computing, its Ethics Board granted approval for the conduct of the research presented in this manuscript with the permit number 251-67/311-25/156, 25 ^th^ April 2025.

Ethics approval was not sought prior to the commencement of this study because the activities were designed as public engagement and educational demonstrations involving non-invasive interactions with robotic platforms. Participation was entirely voluntary, no vulnerable populations were targeted, and no personal, sensitive, or identifiable data were collected. The activities involved observation, hands-on interaction with simulation tools, and supervised piloting of an autonomous surface vehicle in a controlled setting, posing no more than minimal risk to participants. Ethics approval was subsequently obtained retrospectively to formally document compliance with institutional and ethical standards. Given the low-risk nature of the study and its focus on science communication and public engagement, retrospective approval was deemed appropriate.

## Data Availability

Two datasets support the findings of this study: Biograd-based dataset Limerick-based dataset Both datasets are available at https://doi.org/10.5281/zenodo.17467444 ^28^ The authors agree to make freely available all data and materials supporting the results or analyses in this paper, under the Creative Commons Attribution 4.0 International (CC BY 4.0) license, which permits unrestricted reuse, distribution, and reproduction provided the original work is properly cited.

## Footnotes

^1^ https://limerick23.oceansconference.org/
