# Supplementary material for: Citizens marine pilots: Increasing autonomous surface vehicles navigation capabilities with and for the public
Source: Open Res Eur. 2026 Jul 28;6:21. Originally published 2026 Jan 12. [Version 2] doi: 10.12688/openreseurope.21252.2 (PMC13612996; doi:10.12688/openreseurope.21252.2)
Supplement: Peer review history

# Reviewer report: Reviewer response for version 2

**DOI:** 10.21956/openreseurope.26677.r80546

**Published:** 2026-09-24

**Authors:** Siemen Herremans

**Affiliations:** 1 University of Antwerp, Antwerp, Belgium

Summary of article:

 The article presents a report of two citizen science experiment at two events in 2023.

 At the first event, users were asked to navigate 5 scenarios according to the COLREG regulations, and their performance was compared to an autonomous navigation algorithm. In addition, some effort was made in assessing the perceived difficulty of the task.

 At the second event, students were tasked to navigate a remotely controlled USV. This data was then used for imitation-learning purposes.

 Summarizing review:

 I believe the work can be a beneficial contribution to the community, specifically demonstrating a possible way to set up human experiments in this context. There are however some reservations about how the work is presented that should be addressed before I can recommend acceptance.

 Necessary changes before acceptance:

 Limerick experiment:

- It must be made clear if the humans were instructed to take the most energy-efficient path that avoids collision, similar to the autonomous colav algorithm. This is relevant in interpreting the results. If they were not instructed this, explain how this affects the results (humans might have taken a better path otherwise). Clarify this in the work.
- Details must be provided about the dynamics of the vessel: which model from MARUS was used, how many degrees of freedom are there? Is there a sway-yaw coupling? These are relevant to get a feeling about the complexity for the human.
- Details must be provided about the autonomous colav algorithm. In addition, the paper must motive the reasoning behind considering this optimal (this also links to the previous point about if humans got the same instructions).
- The authors must either motive their choice of RMSE over a more interpretable mean-absolute error (MAE), or change figure 4 to MAE.
- Explain the claim that participants were positive, encouraging and highly appreciating, are their responses recorded and available? Consider removing this claim if it cannot be motivated.
- Review the work to remove or back-up unscientific claims, such as "marine robotics creates the most interdisciplinary and participatory scientific pool", "it is now more important than evert to disseminate ...", "but also played a crucial role in fostering social acceptance of autonomous robots", "in a healthy, competitive atmosphere". These types of claims should be avoided in a scientific work, unless they are motivated by sources or research.

Kumbor experiment:

- No, strictly necessary changes.

Strongly recommended changes:

- Provide a short comparison of MARUS with other existing simulators and provide references to those.
- Provide a short comparison of Echo State Network with other existing imitation learning approaches in the ocean engineering domain and provide references.
- Explain why an RNN is necessary over a normal feed-forward NN. i,e,. why are the previous outputs relevant to the decision making in this case?
- Acknowledge the rapidly changing motor RPM as a downside (wear and tear on the engine) and highlight some existing tricks to solve this issue.

Minor points: 

- introduced with ROS and with the sensor data -> introduced to ROS and to the sensor data
- On page 9, paragraph 1: explain how participants gained knowledge about controlling ASVs
- Page 10: Explain that x, y and \psi are the positing and yaw of the vessel.
- Explain what is meant by "the executed paths are very similar"

Is the study design appropriate and does the work have academic merit?

Yes

Is the work clearly and accurately presented and does it cite the current literature?

Partly

If applicable, is the statistical analysis and its interpretation appropriate?

Partly

Are all the source data underlying the results available to ensure full reproducibility?

Yes

Are the conclusions drawn adequately supported by the results?

Partly

Are sufficient details of methods and analysis provided to allow replication by others?

Partly

Reviewer Expertise:

Autonomous vessel navigation, 3D simulators, AVS simulation, AI, robust machine learning, reinforcement learning.

I confirm that I have read this submission and believe that I have an appropriate level of expertise to confirm that it is of an acceptable scientific standard, however I have significant reservations, as outlined above.

# Reviewer report: Reviewer response for version 2

**DOI:** 10.21956/openreseurope.26677.r80539

**Published:** 2026-09-24

**Authors:** Anas S. Alamoush

**Affiliations:** 1 World Maritime University, Malmö, Sweden

Anas Alamoush

 World Maritime University, Malmö, Sweden

 The paper reads well and the outreach work is worth reporting. A few things need fixing before I can approve it:

- The title claims the work increases ASV navigation capabilities. It does not show that. What we have is one AI-driven path compared with one skilled pilot, with no numbers. Either add a measurable result for the trained controller or bring the title and abstract down to what was done.
- The Kumbor activity has no results on the participants. Twenty-five students drove the vehicle, but we do not know how many runs went into training, how they were chosen, or what the students got out of it. A short survey like the one in Limerick would have made the two activities comparable.
- The Limerick numbers do not line up: 12 attendees, then 13/12/11/11/11 trials, then 20 survey respondents. Say clearly who did what. Also, 16 of the 20 came from a robotics tutorial at OCEANS, so calling them the general public is a stretch.
- Scoring players only on deviation from the algorithm's path punishes anyone who kept a safer distance. This needs a sentence or two in the results.
- Some points raised in the first round are still open: "digital twin" is still in the Conclusions, and the passing-distance issue is not discussed.
- The ethics statement says no vulnerable groups were involved, yet the Kumbor participants were high-school minors. Please reconcile this.
- Data availability mentions a Biograd dataset, but no Biograd activity appears in the paper.
- The Introduction refers to Section 1 and Section 2, but the headings are not numbered.
- The Conclusions mostly repeat the activities. Two or three sentences on what the authors learned and would do differently next time would add more value.

Competing Interests: No competing interest exists

Is the study design appropriate and does the work have academic merit?

Partly

Is the work clearly and accurately presented and does it cite the current literature?

Partly

If applicable, is the statistical analysis and its interpretation appropriate?

Partly

Are all the source data underlying the results available to ensure full reproducibility?

Partly

Are the conclusions drawn adequately supported by the results?

Partly

Are sufficient details of methods and analysis provided to allow replication by others?

Partly

Reviewer Expertise:

Port energy transition, technology and innovation

I confirm that I have read this submission and believe that I have an appropriate level of expertise to confirm that it is of an acceptable scientific standard, however I have significant reservations, as outlined above.

# Reviewer report: Reviewer response for version 2

**DOI:** 10.21956/openreseurope.26677.r80540

**Published:** 2026-09-24

**Authors:** Chanaka Thushitha Bandara

**Affiliations:** 1 University of Delaware, Newark, DE, USA

This paper presents two citizen science activities carried out under the EU-funded MONUSEN project, aimed at engaging the general public with marine robotics and autonomous surface vehicles. The first activity, held at OCEANS 2023 in Limerick, invited participants to navigate a simulated vessel through collision avoidance scenarios using the MARUS simulator. The second, held at the Breaking the Surface workshop in Kumbor, Montenegro, had high school students manually pilot the SWAMP autonomous surface vehicle, with their control data subsequently used to train a neural network controller via imitation learning. Together, the two activities demonstrate a compelling model for integrating citizen engagement into robotics research, simultaneously advancing scientific goals and fostering public awareness of autonomous marine systems.

 The paper is well-written and the research goals are clearly motivated. The dual focus of using citizens to generate training data while also educating them is an elegant framing that gives the work both scientific and societal value. The revised version has addressed the prior reviewers' comments thoughtfully, including clarification of terminology regarding main ship and target ship, improvements to the definition of the optimal path, better figure readability, and a more nuanced treatment of the IMO regulatory landscape for autonomous vessels. However, sufficient details of methods and analysis for full replication are only partly provided. While the imitation learning paradigm is explained with reference to Odetti et al. 2020 and the Zenodo dataset is publicly available, several methodological details remain underspecified. In particular, it is not clearly stated how many training runs per student were recorded in the Kumbor activity, nor how the CNR team selected which trajectories were used for training versus validation. For the Limerick activity, it remains unclear whether the target ship behavior in each game level was scripted or algorithmically driven, which is a detail that would be necessary for anyone seeking to reproduce the experimental setup. The authors are encouraged to address these points explicitly in the methods section to bring the paper to a standard that would allow independent replication.

Is the study design appropriate and does the work have academic merit?

Yes

Is the work clearly and accurately presented and does it cite the current literature?

Yes

If applicable, is the statistical analysis and its interpretation appropriate?

Yes

Are all the source data underlying the results available to ensure full reproducibility?

Yes

Are the conclusions drawn adequately supported by the results?

Yes

Are sufficient details of methods and analysis provided to allow replication by others?

Partly

Reviewer Expertise:

Marine robotics, controls, reinforcement learning, autonomous surface vehicles, imitation learning, human robot interaction, and marine simulation and verification.

I confirm that I have read this submission and believe that I have an appropriate level of expertise to confirm that it is of an acceptable scientific standard.

# Reviewer report: Reviewer response for version 1

**DOI:** 10.21956/openreseurope.22986.r74684

**Published:** 2026-06-22

**Authors:** Lovro Maglić

**Affiliations:** 1 Nautical Sciences, Faculty of Maitime Studies, University of Rijeka, Rijeka, Croatia

The article is interesting and clearly presented. The methods and analysis are sufficiently detailed, and the results are reproducible. However, several minor details should be addressed as follows:

- In the introduction section, sixth paragraph, there is a statement saying that the operation of autonomous marine vehicles currently lacks regulations by the IMO. This statement is too broad and general because there are regulations for MASS ships issued by the IMO. I suggest that you present what the IMO has done until now, and what the authors think is lacking in the regulatory framework.
- On page 4, first paragraph in the results section, it is not clearly defined what the main goal was. I suggest that the authors, at the beginning of the section, explain that the path of the autonomous algorithm is considered optimal (maybe presented as a target path so that players try to match the path as much as possible?). This should also clarify the “best and the worst performing player” in the following paragraph. Otherwise, one could think that the goal is not to make contact, while the seafarers also consider the minimum acceptable distance from vessels while maneuvering.
- In the same section, it is not defined what is considered a “target ship” and “main ship”. I suggest you state which is a “give-way” and “stand-on” ship.  

Is the study design appropriate and does the work have academic merit?

Yes

Is the work clearly and accurately presented and does it cite the current literature?

Yes

If applicable, is the statistical analysis and its interpretation appropriate?

Yes

Are all the source data underlying the results available to ensure full reproducibility?

Yes

Are the conclusions drawn adequately supported by the results?

Yes

Are sufficient details of methods and analysis provided to allow replication by others?

Yes

Reviewer Expertise:

Transport technology, Safety at sea, Marine pollution, Marine technologies, Port organization.

I confirm that I have read this submission and believe that I have an appropriate level of expertise to confirm that it is of an acceptable scientific standard.

# Response

**Published:** 2026-07-15

**Authors:** Simona Aracri

**In the introduction section, sixth paragraph, there is a statement saying that the operation of autonomous marine vehicles currently lacks regulations by the IMO. This statement is too broad and general because there are regulations for MASS ships issued by the IMO. I suggest that you present what the IMO has done until now, and what the authors think is lacking in the regulatory framework.**

*Thank you for this observation. We agree that the original statement was overly broad and did not adequately reflect recent developments in the regulatory framework for autonomous vessels. To address this comment, we revised the Introduction to acknowledge the significant progress made by the International Maritime Organization (IMO) regarding Maritime Autonomous Surface Ships (MASS). In particular, we now note that the IMO adopted the International Code of Safety for Maritime Autonomous Surface Ships (MASS Code) in May 2026, representing an important step toward the integration of autonomous vessels into the maritime domain and the establishment of dedicated operational regulations. We further clarify that our concern is not the absence of regulation for autonomous shipping in general, but rather the limited coverage of the rapidly growing ecosystem of smaller and unconventional aquatic robotic platforms, including autonomous surface vehicles used for research, environmental monitoring, and citizen-operated applications. These systems are only partially addressed by current regulatory frameworks, creating a gap between technological innovation and regulatory development. The revised text now better distinguishes between the progress achieved for MASS and the remaining challenges associated with emerging autonomous marine platforms.*

**On page 4, first paragraph in the results section, it is not clearly defined what the main goal was. I suggest that the authors, at the beginning of the section, explain that the path of the autonomous algorithm is considered optimal (maybe presented as a target path so that players try to match the path as much as possible?). This should also clarify the “best and the worst performing player” in the following paragraph. Otherwise, one could think that the goal is not to make contact, while the seafarers also consider the minimum acceptable distance from vessels while maneuvering.**

*Thank you for this valuable comment. We agree that the objective of the experiment was not sufficiently clarified in the original manuscript. To address this, we have revised the beginning of the Results section to explicitly state that the trajectory generated by the autonomous navigation algorithm is considered the reference (target) path. Participants were instructed to navigate the vessel while following this path as closely as possible, while still applying their own judgment and experience regarding safe maneuvering practices. We have also clarified that the "best" and "worst" performing players are identified based on their deviation from the reference trajectory, rather than solely on proximity to other vessels or collision avoidance outcomes. We acknowledge that professional seafarers naturally consider minimum acceptable passing distances and other safety-related factors when maneuvering. This point is now discussed in the manuscript to better contextualize the observed differences between human and autonomous navigation strategies.*

**In the same section, it is not defined what is considered a “target ship” and “main ship”. I suggest you state which is a “give-way” and “stand-on” ship.**

*Thank you for this comment. We agree that the terminology was not sufficiently defined in the original manuscript. To improve clarity, we have revised the text to explicitly define the main ship as the MARUS vessel, i.e., the vessel controlled by the autonomous collision avoidance algorithm. The term target ship refers to any surrounding vessel involved in a potential encounter with the main ship. Depending on the scenario, the encountered vessel acted either as the stand-on vessel or the give-way vessel according to the International Regulations for Preventing Collisions at Sea (COLREGs). Where relevant, we have clarified the respective roles of the vessels in the considered scenarios. These definitions have been added at the beginning of the Results section and are used consistently throughout the manuscript.*

# Reviewer report: Reviewer response for version 1

**DOI:** 10.21956/openreseurope.22986.r68430

**Published:** 2026-03-25

**Authors:** Emerson Martins de Andrade

**Affiliations:** 1 Federal University of Rio de Janeiro, Rio de Janeiro, Brazil

Concerning the specific comments below, I would ask the authors to **clarify** them **in the manuscript** ( **i.e., giving more information, describing in a clear way; etc.**).

1. Abstract: …closer-to-reality V&V…, define V&V.
2. “…OCEANS 2023 Limerick, We presented…”, change “We” to lowercase.
3. When the authors state: “Figure 1 shows the paths of the main ship, for the 5 different scenarios, when controlled by an autonomous collision avoidance algorithm.” Is this an autonomous or automated process? Maybe some more information on that could be beneficial.
4. Considering that the main ship is controlled with the autonomous collision avoidance algorithm, which provides the “optimal” path for each case, it will be interesting to clarify what is “optimal” here, for instance, is the optimal considering the energy perspective? some safety distance? shortest path? …
5. The mentioned RMSE computation is considering which variable? The path (X, Y)? The error between the paths? And about the time (they are probably not synchronized)? Also, why consider only the RMSE as a metric?
6. All text in Figures 1 to 5 is too small; I would suggest increasing it to improve readability.
7. Figs 1 to 5: I would suggest adding something like (A), (B), (C), etc for each case. For instance, if we consider Fig. 4, we can not say which scenario each subplot represents. Also, the authors state the quantitative results based on 12 attendees, but Fig. 4 shows only 11 (except for the first subplot).
8. Fig. 5 does not have units.
9. In the text, we have: “… procedure, the reader is referred to ^26^ …”, is this citation format correct?
10. In the illustration of Figure 8, is there no input for environmental parameters? Wind can be a problem considering the lateral windage area of the USV. Also, the currents may affect the USV response, considering its size.
11. Page 9, we have: “…train an AI controller …”, define AI.
12. Suddenly, in the conclusions, a “digital twin” is mentioned. As I understood, you have a simulator, not a digital twin. Personally, did not get the point here.
13. “In a healthy, competitive, atmosphere, students gained an understanding of…”, the comma after competitive seems incorrect.

Is the study design appropriate and does the work have academic merit?

Yes

Is the work clearly and accurately presented and does it cite the current literature?

Yes

If applicable, is the statistical analysis and its interpretation appropriate?

Partly

Are all the source data underlying the results available to ensure full reproducibility?

Yes

Are the conclusions drawn adequately supported by the results?

Yes

Are sufficient details of methods and analysis provided to allow replication by others?

Yes

Reviewer Expertise:

Robotics, Ocean Engineering

I confirm that I have read this submission and believe that I have an appropriate level of expertise to confirm that it is of an acceptable scientific standard, however I have significant reservations, as outlined above.

# Response

**Published:** 2026-07-15

**Authors:** Simona Aracri

**Abstract: …closer-to-reality V&V…, define V&V.**

*Thank you we added*

**– Verification & Validation –.   “…OCEANS 2023 Limerick, We presented…”, change “We” to lowercase.**

*Thank you, corrected*

**When the authors state: “Figure 1 shows the paths of the main ship, for the 5 different scenarios, when controlled by an autonomous collision avoidance algorithm.” Is this an autonomous or automated process? Maybe some more information on that could be beneficial.**

*Thank you for this comment. We agree that the wording could be clarified. In this context, the process is autonomous in the sense that the main ship is controlled by the collision avoidance algorithm which is autonomous. We modified the caption of Figure 1as follows “Shows the paths of the target ship and the main ship generated by the autonomous collision avoidance in the following scenarios (left to right): Overtaking, Head on, Right crossing, Left crossing, Head on - Right crossing.”*

**Considering that the main ship is controlled with the autonomous collision avoidance algorithm, which provides the “optimal” path for each case, it will be interesting to clarify what is “optimal” here, for instance, is the optimal considering the energy perspective? some safety distance? shortest path? The text says that the optimal situation is the collision avoidance and fuel saving**

*Thank you for this valuable comment. In this work, the term optimal refers to the trajectory generated by the autonomous collision avoidance algorithm that simultaneously satisfies the collision avoidance requirements while minimizing fuel consumption. Now the relevant sentence reads: “The same collision situations are tested with an autonomous ship and autonomous collision avoidance algorithms. The additional goal is to compare the efficiency of citizens’ choices and with those generated by the autonomous collision avoidance algorithm, where the algorithm computes trajectories that ensure collision avoidance while minimizing fuel consumption. This comparison aims to inform citizens of the importance of making navigation decisions that are both safe and fuel-efficient, thereby contributing to energy savings and reducing the environmental impact of maritime operations.” * 

**The mentioned RMSE computation is considering which variable? The path (X, Y)? The error between the paths? And about the time (they are probably not synchronized)? Also, why consider only the RMSE as a metric?**

*We thank the reviewer for this comment. The RMSE is computed on the 2D position error between each participant's traversed path and the reference (optimal) path generated by the autonomous path planner. Specifically, for each pair of paths, both are resampled to a common number of points by normalized path progress (fraction of trajectory completed) and the RMSE is calculated as the root-mean-square of the point-wise Euclidean distance between them. We note that this correspondence is established by path progress rather than by elapsed time, since paths of different duration cannot be directly time-aligned, we acknowledge this as a limitation of the current metric, as it does not capture timing differences between participants and the reference trajectory.*

**All text in Figures 1 to 5 is too small; I would suggest increasing it to improve readability.**

*We thank the reviewer for this comment. We have revised Figures 1 to 5 to substantially increase the font size of all axis labels, tick labels, legend text, and panel titles, improving overall readability. The updated figures have been regenerated accordingly.*

**Figs 1 to 5: I would suggest adding something like (A), (B), (C), etc for each case. For instance, if we consider Fig. 4, we can not say which scenario each subplot represents. Also, the authors state the quantitative results based on 12 attendees, but Fig. 4 shows only 11 (except for the first subplot).**

*We thank the reviewer for this suggestion. We have added a descriptive label to each subplot in Figures 1 to 5 identifying its corresponding scenario (e.g., "1 - Overtaking"), which resolves the ambiguity noted for Figure 4. Regarding the sample size: not all participants completed every scenario, so the number of valid trials varies across scenarios. The per-scenario sample sizes shown in Figure 4 are 13, 12, 11, 11, and 11 for scenarios 1 through 5, respectively, rather than a fixed 12 in every case. We have clarified this in the manuscript text to avoid the apparent inconsistency.  *  

**Fig. 5 does not have units.**

*We thank the reviewer for this comment. The deviation shown in Figure 5 is the point-wise Euclidean distance (in meters) between each participant's path and the reference path. We have updated the y-axis label to "Deviation [m]" to make the units explicit.*

**In the text, we have: “… procedure, the reader is referred to ^26^ …”, is this citation format correct?**

*Thank you for pointing this out, we modified the sentence to: “. For a detailed description of the neural controller and the training procedure, the reader is referred to the manuscript Odetti et al. 2020 ^26^”*

****In the illustration of Figure 8, is there no input for environmental parameters? Wind can be a problem considering the lateral windage area of the USV. Also, the currents may affect the USV response, considering its size.**

*Thank you for this observation. The control architecture illustrated in Figure 8 only includes the joystick input, as the figure is intended to represent the user control interface rather than the complete dynamic model of the USV. Environmental disturbances, such as wind and currents, are therefore not included as inputs in this illustration. * 

**Page 9, we have: “…train an AI controller …”, define AI.**

*Thank you for pointing this out. We have addressed this in the revised manuscript by defining AI as Artificial Intelligence at its first occurrence.*

 S **uddenly, in the conclusions, a “digital twin” is mentioned. As I understood, you have a simulator, not a digital twin. Personally, did not get the point here.**

*Thank you for this comment. We agree that the use of the term digital twin was not appropriate in the context of this work. Where relevant, we have removed the digital twin wording and replaced it with terminology that more accurately reflects the simulator used in the study.*  

**“In a healthy, competitive, atmosphere, students gained an understanding of…”, the comma after competitive seems incorrect.**

*Thank you for pointing this out. We have corrected the punctuation by removing the unnecessary comma **.***
